# American Dental Association

**Published:** 1860-06

**Authors:** 


### AMERICAN DENTAL ASSOCIATION.

Messrs. Editors:—As the time proposed for the formation of the above named Association draws nigh it may be well to suggest a few thoughts concerning the "Plan of Organization" submitted, through the journals, by the Committee appointed for the purpose at the Niagara meeting.

From the report of this meeting, as published in the *Dental Cosmos*, it appears that this Committee was appointed on the evening of August 3d, and reported on the afternoon of the next day. Though the Committee is composed of men of undoubted talent, all will agree that the time was much too short to admit of a matured, or carefully prepared report, even if there had been nothing to distract or divide the attention. But when it is remembered that the American Dental Convention was in session, that the place was *Niagara*, with all its attractions, and Blondin besides, it would be unreasonable to expect a suitable report from *any* body on such short notice. It seems, moreover, that it was regarded as immature; for on motion of the oldest and most experienced member of the meeting, it was recommitted, "with instructions to *mature*, and amend, if necessary, and publish in the Dental Journals as early as November." It appears, however, that the Committee either thought it needed little or no amendment, or felt themselves incompetent to carry out the instructions. At any rate, they published it immediately, with "but a slight modification."

It is unfortunate that the Committee was required to present its report so soon; for as it was to lie over a whole year, there was no call for haste. True, some may think that, as it was to be subject to revision and correction at the next meeting, it mattered but little what kind of a report was presented. But all such reasoning is fallacious. The farmer can do much neater plowing in an unbroken sod than where numerous strong furrows have been drawn. The builder is much more likely to erect a good structure on a naked lot, than on a defective foundation already laid. On the same principle, a defective, unmatured report, lying on the table, is a serious nuisance to any meeting.

When such a committee has more labor assigned to it than can be properly performed in the stipulated time, it becomes necessary to slight the work, or to *copy* the plan of some other society; unless one of its members has a "cut and dried" article ready for presentation, which would be a fortunate arrangement, if these over-willing *cut and dry* souls were always as competent as they fancy themselves. From the report of this committee, it appears that the copying expedient, at least, was rescrted to; and it is quite probable, the "cut and dry" was coupled with it. The plan submitted "is based upon a constitution that was framed by some of the brightest and best minds of our country," we are told, which may be all true; and these "best minds" may have framed a very good constitution, but it does not follow that it is, in any way, adapted to the wants of a dental association. While Smith is corpulent, and Jones is slim, Smith's coat will not fit Jones, even though "framed by some of the brightest and best (tailors) of our country." We are reminded of the school boys who, on being appointed to draft a constitution for a "*Boy's Debating Club*," presented the rules and regulations of the "*Society for the Relief of Indigent Lying in Women*," and insisted on their adoption, on the ground that their mothers, who framed these rules, knew a great deal more about societies than they did.

But the report is as good as can be expected from the time allowed to the Committee. They might have done more however, in the way of amending, when it was referred back to them; but it is usually much easier to prepare a new document, than to perfect a seriously defective one. Under all the circumstances it is little wonder that the report is verbose and ambiguous, that it frequently "sets the rules of grammar at defiance," and ignores the science of rhetoric. The Committee had not *time* to make a concise and accurate report, much less to consult and deliberate as to the great wants of the profession in reference to a new national organization.

But the document is now before the profession; and it may become necessary to make the best of it. It is composed of six sections, regularly named and numbered, and two additional divisions or sections, *not numbered.* The boy's excuse for not counting the little spotted pig was that it wouldn't keep still enough. The reason for not numbering here is less obvious.

The first section treats of the NAME, and declares that "This organization shall be known by the name, style and title of the *American Dental Association.*" Now, though good taste would require the omission of "style and title," yet, as it is important to let the world see that dentists can use a good many words, it would be well to retain them, and supply, in addition, the words, *appellat'on*, *denomination*, and *cognomen*, as likewise nearly synonymous with name.

The third section refers to MEMBERS, and proposes to have *delegates*, *members* by *invitation* and *permanent members*, all to have the same powers and privileges. The members by invitation are to be reputable practitioners from sections of the country not otherwise represented. They are to "receive their appointment by invitation of the meeting." This is certainly very indefinite, and liable to great abuse. As few in the profession have the firmness to vote against the invitation of a brother who is present, even though they regard him as not worthy of membership, many are likely to be admitted that will not be desirable members. There can be no objection to inviting such a set as consultative members; but to allow them to "vote on all questions" is carrying the matter of courtesy a little too far.

There are two provisions for becoming "permanent members,"—by receiving a unanimous vote of the meeting, and by being appointed a delegate once in a lifetime; for we are told that these "shall consist of all those who have served in the capacity of delegates." Now, let us adopt this, and we who have the good fortune to be delegates at the first meeting can, ordinarily, control the affair ever afterwards. What if our constituents do not approve of our course? They can not set us aside. True, they can elect other delegates; but we can go with them, and, at least, tie their votes. We will have all the advantages of experience, while they will be but fresh recruits. I rather think we will all vote for this clause; for who is such a fool that he will not vote himself into a place of power, when he has an opportunity to do it? But, seriously, if it is the intention to admit any but delegates to the full membership, it is folly to waste time in effecting an organization.

The fifth section refers to OFFICERS. "They shall be nominated by a special committee of one member from every State represented at the meeting, and shall be elected by vote on general ticket."

Who is to appoint this special committee? The President? If so, why not give him the power *directly* to select the officers for the ensuing year, for by this arrangement he can control the matter as certainly, even though indirectly? And if the Association must elect those nominated by the special committee, (and there is no provision for any other candidates,) where is the call for a "vote on general ticket," or any other way? Why not recognize the decision of the committee as final, without the farce of an election under such circumstances?

Only the ordinary officers of an association are proposed; yet we have a long series of clauses defining their duties, just as if no other society ever existed, and just as if custom and parliamentary usage had not definitely fixed the ordinary duties of these officers. "*The President* shall preside at the meetings—.'' Why, of course he shall! Would he be a *president* if he did not preside? And where would he preside but "at the meetings?" "In the absence of the President, one of the Vice Presidents shall assume all the duties of the office." Well, what else is he for? and would he be a *Vice* President if he did not? We are just as gravely told that "The *Corresponding* Secretary shall attend to the *correspondence*—," and that "The Recording Secretary shall keep accurate minutes of the proceedings—," which is certainly as satisfactory and self-evident as that "Bakers bake bread and cakes," or that

"A sea-horse is a sea-horse, when
You see him in the sea."

Now, there is, perhaps, nothing sinful in such verbosity; yet, as the credit of the profession is at stake, it should not be tolerated in a document so highly representative in its character as the constitution of the "American Dental Association." Our brethren across the water sometimes allude to American verbosity. Shall we afford them so strong an occasion to do so again?

The sixth section treats of *Standing Committees.* Of the Committee of Arrangements, one member, "if possible," is to "reside in the place at which the Association is to hold its next ensuing annual meeting." Now, if there is any place where it would be *impossible* for one member to *reside*, it would be impracticable for a whole association to *sojourn* there long enough to hold a meeting. If the Committee had taken more time to "mature and amend," when the document was referred back, they would have said, *if practicable*, instead of "if possible;" and they would have corrected a great many other expressions, of which this may serve as a sample.

Then, there is a double-geared standing committee on "Scientific Investigation," one-half of which is to institute a series of investigations and experiments on the "Physiological and Microscopical Anatomy of the Teeth," while the other half is to undergo similar labors with reference to "Chemistry in its relations to Dentistry," and each half, separately, (or both recombined, it is hard to tell which) is to "present to the Association the result of their labors." Why these *two committees*, on subjects so distinct from each other, are ranked together as one, it is hard to see. It would be just as consistent, and quite as convenient, to have but one standing committee, and subdivide it so as to embrace all the duties provided for by the seven proposed in the report.

Another one is called "The Committee on Dental Pathology and Surgery," and is required to take "under consideration every thing that appertains to the pathological conditions of the teeth," etc., and to thoroughly test all improvements in Operative Dentistry. But it would seem that this "consideration" and *testing* are not to be conducted *scientifically*, for the preceding one is the "committee on *scientific* investigation." Moreover, this committee is to report "*before* the Association;" but who they are to report to, or how long before, is not told.

But it is unnecessary to specify farther. No members of the profession know better than the committee that the document needs thorough and careful revision; and, as they have cordially and frankly invited the profession "to consider and remedy its defects"—to remedy its *imperfections* and *shortcomings*—these remarks, offered with the kindest feelings, will not be regarded as out of order. E. T.

